# Undergoing radical treatment for prostate cancer and its impact on wellbeing: A qualitative study exploring men’s experiences

**DOI:** 10.1371/journal.pone.0279250

**Published:** 2022-12-16

**Authors:** Neel Vyas, Oliver Brunckhorst, Louis Fox, Mieke Van Hemelrijck, Gordon Muir, Robert Stewart, Prokar Dasgupta, Kamran Ahmed

**Affiliations:** 1 MRC Centre for Transplantation, Guy’s Hospital Campus, King’s College London, King’s Health Partners, London, United Kingdom; 2 Translational Oncology and Urology Research (TOUR), School of Cancer and Pharmaceutical Sciences, King’s College London, London, United Kingdom; 3 Department of Urology, King’s College Hospital, London, United Kingdom; 4 King’s College London Institute of Psychiatry, Psychology and Neuroscience, London, United Kingdom; 5 South London and Maudsley NHS Foundation Trust, London, United Kingdom; 6 Urology Centre, Guy’s and St. Thomas’ NHS Foundation Trust, King’s Health Partners, London, United Kingdom; 7 Department of Urology, Sheikh Khalifa Medical City, Abu Dhabi, United Arab Emirates; 8 Khalifa University, Abu Dhabi, United Arab Emirates; Caleb University, NIGERIA

## Abstract

**Introduction:**

Quality of life in prostate cancer survivorship is becoming increasingly important, with mental and social wellbeing recognised as key components. However, limited global evaluation of psychosocial challenges experienced after treatment exists. Therefore, we aimed to explore the lived experiences of men who underwent radical treatment, and its psychosocial impact.

**Material and methods:**

This qualitative study was conducted using 19 men who had undergone radical treatment (prostatectomy or radiotherapy) for their cancer. Semi-structured interviews were conducted exploring lived experiences of men after treatment. A Structured thematic analysis of collected data was undertaken, with an inductive co-construction of themes through the lens of the biopsychosocial model. Themes generated were considered within a psychological, social, and physical wellbeing framework.

**Results:**

An initial knowledge gap meant mental wellbeing was strongly impacted initially leading to a ‘Diagnostic Blow and the Search for Clarity’. Doubt over individuals’ future resulted in ‘An Uncertain Future’ in many men. Once treatment was completed a ‘Reflective journey’ began, with men considering their outcomes and decisions made. Social wellbeing was also impacted with many identifying the ‘Emotional Repercussions’ on their relationships and the impact their diagnosis had on their partner and family. Many subsequently sought to increase their support through ‘The Social Network and Advocacy’, while physical changes led to an increased need for ‘Social Planning’. Finally, physical wellbeing was highlighted by a continual acknowledgement of the ‘Natural process of ageing’ leading to a reluctancy to seek help, whilst simultaneously attempting to improve existing health via ‘The Health Kick’.

**Conclusions:**

Radical treatments have a considerable impact on mental and social wellbeing of individuals. Anxiety after diagnosis and significant uncertainty over individual futures exist, with physical complications of treatment leading to social repercussions. Future research should aim to identify forms of support to improve quality of life of these men.

## Introduction

Prostate cancer remains is the second most common cancer in men representing a large public health problem [1]. Combining this with high and improving survival rates means that increasing number of men are now living with and beyond their disease [2]. With this has come the growing realisation that living longer doesn’t always equate to good quality of life and wellbeing. Long-term physical consequences are well documented, including urinary and sexual dysfunction [3]. However, more recently the psychosocial impact of disease is becoming more apparent. Depression and anxiety are common, with an estimated 30–50% of prostate cancer patients having associated psychological problems irrespective of stage and progression of the cancer [4, 5]. Similarly, the social impact of disease and treatment are significant, having consequences on relationships with partners, wider family, and friends, leaving men often feeling isolated [6]. This recognition has led to numerous survivorship initiatives including the National Cancer Survivorship Initiative (NCSI) in the United Kingdom and similar programmes across the United States including the American Cancer Society (ACS) and the American Society of Clinical Oncology (ASCO), highlighting the growing importance of considering these issues in prostate cancer care [7, 8].

Quality of life and wellbeing in prostate cancer care can be considered through the biopsychosocial model of health which has been refined since its inception in the 1980s [9]. This allows for an understanding of a patient’s subjective experience and its contribution towards diagnosis and healthcare outcomes, meaning individuals can be considered as individuals in a social framework with a specific attention to subjective experience of cancer in the context with clinical data [10]. This has resulted in a shift in healthcare from focusing on disease specific care to a more patient-centred approach. It highlights the relationship between the physical and psychological aspect of health is complex and that subjective experience do not always result from physiological causes. Therefore, consideration of all biological, psychological and social contributions can allow a holistic view into a patient’s wellbeing [11].

When considering mental wellbeing for patients with prostate cancer, further issues exist beyond anxiety and depression which are often overlooked. Fear of cancer recurrence and PSA Anxiety is common and is associated with functional impairment, reduced quality of life and behaviours demonstrated to seek reassurance [12, 13]. This has also been highlighted as one of the most common unmet cancer needs [14]. Additionally, issues surrounding masculinity and body image appear important in this population and represent constructs which assess the concept of self-worth representing the view of our body and mind and how it is perceived by others around us [15]. Studies have demonstrated that men who experience physically apparent changes (e.g. hot flushes) exhibited a loss in identity with reports of a reduction in self-esteem, self-loathing and demasculinization [16].

Similarly, it is important to consider defining elements of social wellbeing in prostate cancer. Social wellbeing can be defined by five key dimensions: integration, contribution, coherence, acceptance and actualisation [17]. In this study, we consider social wellbeing as the relationships between individuals and their partner, family, friends and wider community [18]. Additionally, the individuals daily functioning including occupation, hobbies, and interests. Major impacts of social distress can lead to problems in relationships and support, restriction in activities and challenges of work and occupation [19].

Whilst there is evidence to demonstrate the impact on prostate cancer patients’ wellbeing, there remains a strong quantitative focus in the literature likely underestimating this impact [20]. Additionally, these quantitative approaches use pre-structured questionnaires that can’t be individualised, and therefore don’t capture all experiences and tend to ignore mental and social wellbeing in favour of physical health [21, 22]. Additionally, radical treatment treatments for prostate cancer result in significant physical sequalae such as urinary and sexual dysfunction which themselves are linked to psychosocial issues including changes in partner relationships or masculine self-esteem [6, 15]. However, many qualitative studies combine treatment groups, including those undergoing surveillance and hormone monotherapy, thereby ignoring these unique issues. Lastly, the evidence that does exist for radical treatment is often focused on evaluating specific constructs like masculinity, meaning little global evaluation exists of patients lived experiences and important psychosocial wellbeing constructs. These highlight the need for further qualitative research exploring men’s experiences, to provide a holistic and in-depth account which can be used to better support them. Therefore, we aimed to qualitatively explore the lived experiences of men with prostate cancer undergoing radical treatment options with a focus on describing the effect this has on their psychosocial wellbeing.

## Materials and methods

### Study design

The Standards for Reporting Qualitative Research (SRQR) guidelines were used to form the basis for reporting this qualitative study [23]. Our data collection and analysis procedures were grounded in the philosophy of phenomenology, given that we sought to understand participants’ own lived experiences of undergoing radical treatment for prostate cancer with an interpretive/constructivist paradigm for the analysis to acknowledge individuals’ interpretation of the world around them and their experiences and the researcher as integral to the construction of the data insights [24]. These were additionally encompassed within the modern biopsychosocial model of health previously discussed when framing the experiences of men undergoing radical treatment for prostate cancer on subsequent wellbeing [11]. We gained prospective Health Research Authority (HRA) and NHS Research Ethics Committee (REC) approval for the study (NHS REC Reference Number: 20/SC/0070) with all participants providing written informed consent.

### Participants

We utilised a convenience sampling strategy, recruiting eligible participants who had been diagnosed and were subsequently undergoing follow up at a single tertiary unit in London, UK. Inclusion criteria were adult participants over the age of 18 diagnosed with histologically proven prostate cancer who had previously undergone radical treatment for their cancer with curative intent, including prostatectomy or any type of radiotherapy. Patients who had undergone combination treatments such as those who received neoadjuvant/adjuvant hormone therapy for their radiation therapy or salvage prostatectomy, or radiotherapy were also included. However, those undergoing any type of hormone monotherapy, chemotherapy, active surveillance, or watchful waiting were not eligible. Additionally, non-English participants were excluded due to the unavailability of conducting interviews in other languages. No restrictions were placed on time since treatment or specific oncological characteristics. Recruitment was conducted until data saturation was felt to be reached during data collection and analysis. Data were continuously analysed until no new insights had arisen and existing insights were unchallenged by additional data in 4 successive interviews. At this point the data were deemed to be sufficiently saturated for the aims of the study [25]. Based on our broad study aims, sample specificity and analysis methods this was estimated to be reached at 20 participants, however, this was actually reached after 19 participants [26].

### Data collection

Video-interviews were conducted between December 2021–February 2022, due to safety measures during the COVID-19 pandemic. We conducted interviews using a semi-structured approach to enable a coherent and consistent covering of topics throughout, while ensuring in-depth exploration of wellbeing issues encountered with a nature of flexibility in questions asked [27]. These were conducted using a topic guide based on the biopsychosocial model of health and designed to elicit phenomenological experiences of participants. This was developed through a literature review, piloted through previous studies and refined during data collection based on responses received (S1 Table) [28]. Interviews were carried out by one or two interviewers (NV and OB); a BSc and PhD male students respectively with clinical backgrounds, no previous relationships with the participants and formal qualitative interviewing training prior to the study. Individual interviews were audio recorded and subsequently pseudonymised with interviewer notes taken to supplement this.

### Data analysis

An iterative analysis process began after the first interview. These were transcribed in verbatim and exported onto NVivo 12 software to aid with coding management. We used an inductive thematic analysis based on Braun and Clarke’s approach which was framed through the lens of the biopsychosocial model of health, to generate new findings under widely acknowledged domains of mental, social and physical wellbeing [29]. Two researchers (NV and OB) co-constructed new findings through a process of data familiarisation, code generation followed by theme formulation to report repeated patterns. Our coding frame was developed and subsequently adapted through a repeating process until subsequent dual coding reached a consensus as defined by an intercoder reliability of a Cohen kappa coefficient of over 0.8 on the entire data set (S2 Table) [30]. During analysis, in view of study aims and existing knowledge of the physical impact of radical treatment, physical wellbeing themes were generated focused on their impact on psychosocial wellbeing or the impact on general physical health rather than through specific symptoms experienced (e.g., sexual dysfunction). Regular consensus meetings were held to discuss and refine themes and their context within existing findings. This also aided in maintaining a reflexive approach through a continuous process of questioning previous subject assumptions and discussing reflexive interview diaries maintained [31]. Throughout the study we sought to improve our analytical rigour by using multiple coders, ensuring consistency through development of a coding frame with high intercoder reliability, our regular consensus meetings, and maintenance of an audit log. Additionally, investigator triangulation was used through a research team comprised of varying levels of contact with prostate cancer clinically.

## Results

### Participant characteristics

Of the 19 participants interviewed 8 had previously undergone surgery and 11 radiotherapy. Mean age was 68 years old (range 53–78) with time since diagnosis between 2 and 15 years (Table 1). 89% of the participants were married and retired with 37% working in some capacity. All but one participant were heterosexual with ethnicity distributed as follows: White British (78.9%), Black British (10.5%), Black African (5.3%) and Black British-Caribbean (5.3%) (Table 1). Interview durations were between 25 and 55 minutes (mean 41 minutes).

**Table 1:** Participant characteristics.

Participant Number	Age (Years)	Time since Diagnosis (Years)	Time Since Treatment Completion (Months)	Civil Status	Occupation	Sexuality	Ethnicity	Radical Treatment Received
1	66	15	10	Married	Retired	Heterosexual	White British	Radiotherapy
2	60	3	16	Cohabitation	Retried	Homosexual	White British	Radiotherapy
3	75	2	14	Married	Retried	Heterosexual	White British	Radiotherapy
4	78	8	16	Married	Retired	Heterosexual	White British	Radiotherapy
5	69	2	8	Married	Working	Heterosexual	Black British—Caribbean	Radiotherapy
6	53	2	23	Married	Working	Heterosexual	Black African	Prostatectomy
7	61	3	15	Married	Retired	Heterosexual	White British	Prostatectomy
8	76	6	17	Married	Retired	Heterosexual	White British	Radiotherapy
9	77	2	18	Married	Retired	Heterosexual	White British	Radiotherapy
10	59	2	18	Married	Working	Heterosexual	Black British	Prostatectomy
11	65	2	15	Married	Working	Heterosexual	White British	Radiotherapy
12	54	2	12	Married	Working	Heterosexual	Black British	Prostatectomy
13	68	3	7	Married	Retired	Heterosexual	White British	Radiotherapy
14	72	2	17	Married	Retired	Heterosexual	White British	Prostatectomy
15	67	3	24	Married	Working	Heterosexual	White British	Prostatectomy
16	77	3	6	Married	Working	Heterosexual	White British	Radiotherapy
17	68	2	29	Married	Retired	Heterosexual	White British	Prostatectomy
18	70	4	19	Separated	Retired	Heterosexual	White British	Prostatectomy
19	71	8	83	Married	Retired	Heterosexual	White British	Radiotherapy

### Findings

**Fig 1:**
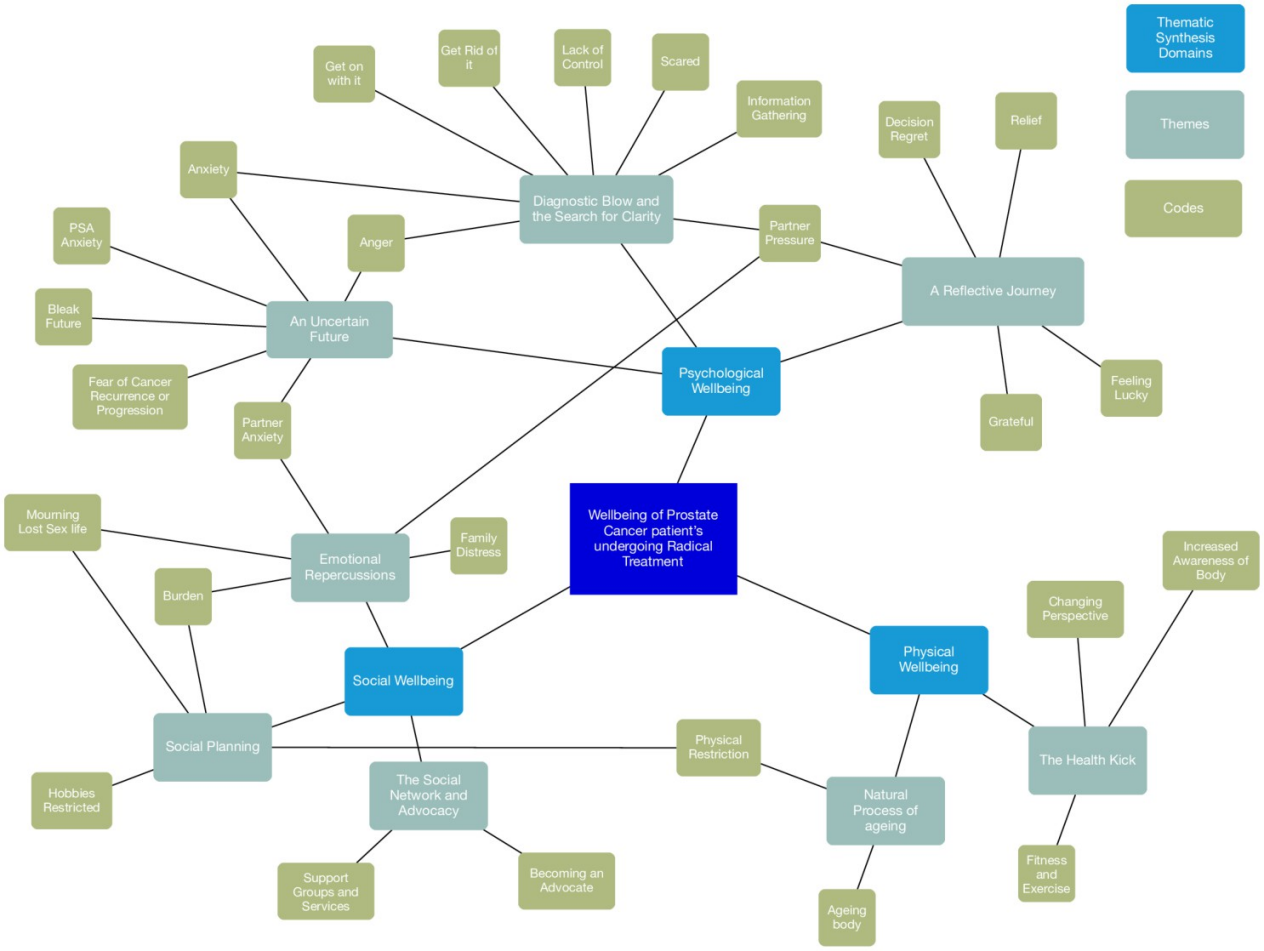
Mind map demonstrating generated themes and selected codes within biopsychosocial thematic domains.

A final 82 codes were generated (S3 Table) leading to a final 8 descriptive themes (Fig 1). Mental wellbeing was the dominant construct generated in coding and led to the themes of ‘*Diagnostic Blow* and *the Search for Clarity’*, *‘An Uncertain Future’* and *‘A Reflective Journey’*. Themes generated for social wellbeing included ‘*Emotional repercussions’*, ‘The *Social network and advocacy’* and ‘*Social planning’*. Finally, under physical wellbeing two themes were generate, the ‘*Natural process of ageing’* and ‘The *Health Kick’*.

### Mental wellbeing

Mental wellbeing contained themes which demonstrated the varied psychological impact experienced by individuals with these demonstrating different stages of this journey and how participants psychological health altered.

#### Diagnostic blow and the search for clarity

When first receiving the diagnosis of prostate cancer, several participants described the feeling of shock, desolation, and despair. Not knowing where to go next and what the future holds. This negatively impacted mood and lead to participants questioning ‘why me?’. Diagnosis was a critical stage of the participant’s prostate cancer journey, from where they observe initial signs of urinary problems and relating those to the idea that this may lead to a prostate cancer diagnosis. This develops as they seek medical professional advice and receive investigation increasing anxiety and leading to the inevitable confirmation that something was indeed wrong. During this diagnostic process many saw the biopsy, and particularly receiving the diagnosis as the worst stage where their feelings of shock, anxiety and low mood often peaked:

*‘I was very*, *very anxious sitting outside waiting to hear because I knew that the biopsy diagnosis was going to be the kind of definite yes or no fork in the road*. *Then I was very anxious going in’*.

*(Participant 7*, *White British*, *age 61)*.

Following on from the initial shock of diagnosis many were left feeling unaware of the treatments available for prostate cancer. There were two distinct approaches to the subsequent decision making of what option is best for them; with some seeking for pragmatic solutions while others hid from the situation. This was often related to the initial reaction, with those struggling to accept the diagnosis often struggling more during their search for clarity.

Overall, most men took a very pragmatic approach upon receiving their diagnosis, including acceptance, and seeking the logical next step. Information gathering formed a big part of this with many seeking reassurances in the form of accessing the internet despite often being aware of the risks of misleading information available on there. However, others sought more personable advice from previous cancer survivors, in particular through hearing their experiences. Most participants took advice from medical professionals and completely trusted this information, particularly those who viewed their diagnosis more as a part of their life journey or those who just wanted to get on with treating it. However, the minority who were less sure about the initial diagnosis were more likely to seek alternate forms of information, particularly through 2^nd^ opinions.

However, some individuals decided to avoid the situation altogether, hoping the problem would evade them and seek for comfort rather than solutions. This perhaps due to the lack of knowledge of the treatment pathways lead to an added worry, a feeling of being overwhelmed and was more common in those who suffered more during the initial diagnosis. This on occasion was shown to delay treatment with participants:

*‘I think at that time I become a little bit anxious about what was going on and when things were going to change*. *So*, *it was around about a year before I started having treatment*.*’*

*(Participant 1*, *White British*, *age 66)*.

#### An uncertain future

Uncertainty surrounding individuals’ circumstances formed a big part of their post diagnostic journey and often lead to a great deal of anxiety once a treatment decision had been made. The source of uncertainty varied significantly. Having made the decision for a specific treatment, an uncertainty surrounding the subsequent outcomes of that treatment was a common source of worry. This was both in terms of successful oncological and functional outcomes. Whilst many understood the risks of urinary and sexual dysfunction, many were still concerned about whether they would have these subsequently, particularly in those undergoing surgery. The weighting of what many perceived as would be better oncological outcomes through surgery, as the surgery would be a definitive removing of the prostate, versus the risk and uncertainty regarding possible urinary incontinence was a core factor in many patients’ decision-making process.

The initial wait for treatment itself proved important for many, and this was more common amongst those undergoing radiotherapy who had to wait several months until their definitive treatment. Combined with this, the Covid-19 pandemic was a key factor that added to the uncertainty of many of the participants’ future. Various participants experienced delays in their surgery or radiotherapy because of this. Being in this unfamiliar situation, several participants were left wondering how long they would have to wait until their definitive treatment began, and if this delay would ultimately impact their disease outcome.

*‘From March until the very beginning of July*. *I didn’t know anything*. *I didn’t know what was going to happen*.*’*

*(Participant 14*, *White British*, *age 72)*.

Once treatment had been completed a new uncertainty surfaced. Whilst many experienced a relief at completing this stage men often worried about whether or not their future would remain cancer free. Whilst often not overtly discussing these issues or stating that they feared recurrence, future hospital appointments, and results of repeat scans and investigations, particularly current PSA values, provided a source of ongoing and repeated stress and anxiety. This demonstrated this repeated fear of cancer recurrence particularly near hospital or doctor interaction points. This appeared particularly common in those who had a stronger initial diagnostic reaction, with individuals who often took a more pragmatic approach, or those who saw their diagnosis as a part of their life journey, experiencing less ongoing worry.

Linked with this was the core role of the PSA test throughout the journey. This was seen as a source of worry among several participants, especially after treatment. A PSA anxiety and obsession of the number at each follow up clinic was commonly demonstrated, despite initially not really understanding what the values meant. With incrementally small increase in PSA would cause huge increases in anxiety and lead to that fear that the cancer may return. This forming a new form of uncertainty once treatment had concluded.

*‘Yeah*, *I mean*, *I think your initial reaction when somebody thought it tells you which is what he did was my peer say*, *eleven point six*. *It’s sort of out of context and you’re thinking and is*, *is that good or is that bad or what does that mean*.*’*

*(Participant 13*, *White British*, *age 68)*.

#### A reflective journey

On completing treatment many reflected on their decisions and treatment with varying satisfaction regarding the outcome it led to. Despite the varying experiences many had, the majority of men were certainly satisfied and ultimately grateful, showing no sense of regret of their individual decision. They were often thankful to staff and departments for giving them their treatment and ensuring that they were appropriately informed to make that decision.

*‘Um*. *Well*, *I was apprehensive at first*. *Um*, *but*, *um*, *thank goodness I went ahead with it because I think it was the right outcome*.*’*

*(Participant 3*, *White British*, *age 75)*.

However, some did exhibit an element of dissatisfaction or decisional regret. Whether to go ahead with surgery or radiotherapy represented a key decision, with some believing that more information and support would have led to them perhaps choosing a different treatment. This unsurprisingly was seen when outcomes had not been as expected. Post-surgery or radiation erectile dysfunction, was a common example of this, often impacting their sexual relationship with their partner. This led them to reflect on regret for undergoing their treatment choice. Additionally, in those undergoing radiotherapy and who had a recurrence there was a belief that having removed the prostate entirely would have prevented this, thereby causing added distress and regret.

*‘Yes*, *that’s exactly what it is*. *That is*, *it is coming back*. *And I suppose*, *again*, *in hindsight*, *I think I benefit from having the operation of having the prostate removed completely*.*’*

*(Participant 1*, *White British*, *age 66)*.

### Social wellbeing

The impact of diagnosis and radical treatment on an individual and their family was often profound. Whilst many maintained and even strengthened relationships with partners, other relationship underwent profound strains with partners and other family members also significantly impacted. Additionally, participants show changes in their daily routine to accommodate the impact of prostate cancer and treatment with many also becoming more socially active through prostate cancer advocacy.

#### Emotional repercussions

Prostate cancer and its treatment had varying implications for individuals’ most important relationship with their partner. Interestingly, most demonstrated a vastly positive change in their relationship, realising they are now more open and transparent with their partner regarding their health and having felt that they had gone through this experience together. An area particularly important was the shared decision making between participants and their partner. This feeling that decisions were not solely their own was critical for some, helping to spread the heavy responsibility with their partners and healthcare professionals.

In line with this, several participants emphasised that diagnosis and treatment of their cancer did not only lead to impacts on their own mental health, but also impacted on the mental health on their partner and family. It appeared in some participants that the anxiety in their partner resulted in more distress than the cancer itself. Therefore, some participants highlighted the need for healthcare staff to consider their partner and family as importantly as themselves when supporting them for mental health in their journey.

*‘You know*, *it’s really hard*, *I think*, *to see your partner*, *lover*, *wife*, *husband suffering or not suffering*, *but having to go through something*.*’*

*(Participant 7*, *White British*, *age 61)*.

However, a small minority did experience an increase strain in their partner relationship with the source of this being vastly varied. Whilst for some the physical implications of disease, including erectile dysfunction took away an element of their relationship. Alternatively, the decision-making process for the treatment was also a source of attrition, with varying views on what treatment options should be undertaken or different views on healthcare professionals themselves examples of these.

*‘Think I felt pressure from my partner as well*, *because I didn’t feel they were being particularly supportive*. *They were trying to be in their way*. *But it wasn’t really being supportive because it was making me question everything that the health profession was trying to do for me*.*’*

*(Participant 2*, *White British*, *age 60)*.

#### The social network and advocacy

A feeling of being within a social network outside the nuclear family of individuals who have undergone, or are undergoing, a similar journey was important for many. Early in the process, speaking to other people through support groups or within their friend group, helped participants feel reassured and further inform their own decision making based on others experiences. Similarly, after treatment this provided a much-needed point to share experiences, to realise physical issues experienced were not alone to them and helped to normalise these. Additionally, some discussed how this was helpful as many felt that others did not understand their experience, and instead weren’t sure how to talk to them about it leaving a small sense of isolation from others.

After experiencing their own prostate cancer journey, many participants increasingly sought to become advocates in their own right. Whilst some sought more formal methods of doing this by becoming involved in support groups themselves or through patient research groups, many did this through more informal means, particularly through promoting the importance of PSA testing to detect cancer early. They share this insight to their friends, family, and wider community, promoting them to get a blood test even if asymptomatic, largely due to the relief of having detected their cancer early through PSA testing.

*‘And if you can get your GP to do a PSA test*, *it’s really well worth doing it*, *you know*, *because it is so symptomatically invisible for such a long time*. *And therefore*, *why wouldn’t you*.*’*

*(Participant 7*, *White British*, *age 61)*.

#### Social planning

The physical repercussions of treatment often had a meaningful impact on participants social lives. Urinary dysfunction, particularly increasing urgency, meant some had a new requirement to plan daily life around the accessibility of a nearby toilet. Whilst this was often not described as a severe limiting factor, this presented an increased thought process to previously simple social activities. Whether this be ensuring the availability of nearby public toilets, or extra stops when planning a long car journey. *‘Yeah*, *sometimes we had to take precautions to make sure there was alternate toileting arrangements if we’re going somewhere difficult*.*’ (Participant 5*, *Black British-Caribbean*, *age 65)*.

Similarly, sexual dysfunction post treatment added an extra need for planning sexual activities with their partners. Whether this be by sildenafil or physical devices such as pumps, the extra step meant there was a loss of spontaneity within their love life and many instead being put off by their use and instead giving up on their love life altogether. However, although some continued to encounter issues and were left with a sense of mourning for their loss of sex life, most did not have a problem at all, with many stating relationships were strong enough to push through this. This was either done by seeking for further solutions for erectile dysfunction or by redefining what intimacy was within their relationship.

*‘The sort of Viagra type of medication which hasn’t really had any impact*. *So*, *the next stage is to have these injections’*.

*(Participant 14*, *White British*, *age 72)*.

### Physical wellbeing

There was a common reluctancy to seek help for the effects of treatment, with many problems often attributed to ageing rather than the treatment itself. However, in reflection, several participants change their views on health and wellbeing and adopt a new healthier lifestyle.

#### Natural process of ageing

Most men attributed the physical sequelae of treatment, particularly sexual and urinary dysfunction as a mere consequence of advancing age. This, as expected was particularly more common in men of older age and who had undergone radiotherapy, with younger men and those who had experienced quicker declines, likely secondary to surgery being less likely to do so.

*‘But I’m in my seventies*. *It’s not as if my sex drive was the same as when I was much younger*.*’*

*(Participant 14*, *White British*, *age 72)*.

*‘But again*, *it feels like old age rather than cancer*.*’*

*(Participant 14*, *White British*, *age 72)*.

This belief was unfortunately detrimental to some. With this attribution, some did not seek help for their urinary symptoms as it was not believed to be something which could be improved and instead must be lived with. Similarly, for sexual dysfunction many instead stated these symptoms were no longer of importance due to their age, and instead drew a line under their sexual life rather than seeking help. Whilst some were happy to do so, due to their age or as a direct trade off for having their cancer treated, many were subsequently left grieving for their lost sex life as a result.

*‘Well*, *obviously*, *I’m not happy about that at all because although I’m seventy-six you know*, *as I say*, *the desire is still very much there*, *but it’s a price you have to pay’*

*(Participant 8*, *White British*, *age 76)*.

*‘Do I miss them*? *Yes*, *sometimes*. *But I’m not sitting there in the corner weeping over the fact of my sex life has disappeared’*

*(Participant 2*, *White British*, *age 77)*.

#### The health kick

Although some participants claimed they were already conscious of their own lifestyle, most participants reflected on changes made because of this experience. Whilst this sometimes occurred directly after diagnosis, whilst waiting for treatment, in the hope of improving outcomes, this was often reported at a later stage. Reflecting on their journey, participants searched for information on lifestyle related risk factors associated with cancer and how they could change to minimize these risks and avoid future disease. This was often achieved through changing their dietary habits, and more commonly increased physical exercise, with these positive changes often appearing to be lasting lifestyle alterations.

*‘You’re more conscientious about things*. *Very much so*. *Yeah*. *Like I said*, *I exercise in the gym three times a week*, *maybe four’*

*(Participant 10*, *Black British*, *age 59)*.

Participants also showed more willingness to go to their doctor to seek for help when problems arose after this experience. Early detection of disease became important so as not to repeat prior experiences, with an increased awareness of their PSA values one example of this. However, this was on occasion unrelated to prostate cancer, with an increased willingness to get any symptoms or issues looked at post diagnosis.

*‘I suspect if you looked at the number of times I got in touch with my GP*, *it would have been very*, *infrequent*. *And I think it’s gone up*. *I think I’ve been much more tuned in*, *perhaps overly tuned in to little signs that there might be something’*

*(Participant 7*, *White British*, *age 61)*.

## Discussion

Quality of life and wellbeing issues in prostate cancer are becoming increasingly important. With patients living longer it is important that they are not just managed physically but are also supported psychosocially. The present study provides an insight into the biopsychosocial implications of radical treatment on men’s experiences after a prostate cancer diagnosis. We demonstrate a global view on patient wellbeing rather than focus on individual constructs, with many prior studies focussing on specific issues such as masculinity [32].

Mental wellbeing was demonstrated as the most significant domain in patient experiences, both in the numerical generation of codes and in richness of data. Receiving the diagnosis was viewed as a critical stage in the prostate cancer journey, with profound anxiety, shock and uncertainty being evident. This initial uncertainty was particularly seen due to the number of different treatment options presented to prostate cancer patients, meaning that whilst future oncological outcomes were good, the resultant quality of life implications were less clear. Due to this uncertainty, information gathering was subsequently a common coping strategy, particularly to explore the different treatment options available, and other individuals experience with these. This uncertainty, continued after treatment, with many ongoing worries about cancer recurrence and treatment outcomes. Lastly, due to the substantial functional load of prostate cancer treatment, on reflection some regretted their treatment choice, however, many instead decided to move on and try to strengthen from the experience.

When considering social constructs many highlighted the emotional impact on their partner and family and their equal need for support. Partner relationships were additionally key in providing a supportive environment with shared decision making and increasing openness commonly seen and only a few describing strains in relationships. Additionally, having a network of support outside the family nucleus was key for seeking reassurance for decision making and to act as advocates. Lastly, planning for physical consequences of treatment and disease also became an ongoing part of individuals daily social life.

Physical health was viewed with contrasting nature. Some patients became more focused on their own health, changing their lifestyle habits and being more likely to seek help for other symptoms. However, commonly patients used ageing as a concept that resulted in side effects experienced and accepted this was part of nature rather than seeking solutions to fix it.

Our findings are in line with existing quantitative literature, indicating anxiety and depression to be higher pre-treatment [33]. This demonstrates the diagnosis and treatment decision-making time to be key, likely not only due to the shock of receiving the diagnosis but also the uncertainty that comes with it in terms of treatment options, timeline, and future outcomes. This uncertainty does not finish upon treatment, with fear of recurrence and PSA anxiety persisting well beyond the initial period. This has previously been demonstrated to be an important aspect of cancer survivorship at various times along the pathway [34]. Uncertainty is therefore important to consider during treatment and survivorship, having substantial implications for mental wellbeing and overall quality of life, and being significantly associated with physical wellbeing [35].

A strong social network was vital for patients to discuss their worries and anxieties to friends and family to relieve the burden of their diagnosis and treatment. Similar findings have been found in other cancers with a strong family support being a strong correlating factor for quality of life and a protective factors against major depressive disorders [36, 37]. Additionally, it has been demonstrated that men with prostate cancer men seek support from those who have undergone similar experiences to them [38]. This means that these a consideration should be given to improve patient social factors, family support and also peer support, offering key ways that could improve mental wellbeing and the patient experience after diagnosis [39].

Interestingly, we found that constructs such as masculinity and body image were of less direct concern in our sample. This is in contrast with major portions of the previous literature where demasculinazation post sexual dysfunction, poorer self-esteem and a loss of body ownership have been highlighted as common issues [15]. This contrast may be due to societal evolving ideas surrounding masculinity with many previous studies conducted several years ago [40]. However, more likely, rather than dismissing these as important issues this highlights the broader approach we took on questioning experiences of wellbeing, which therefore instead demonstrated other important aspects to prostate cancer patients such as uncertainty and fear of recurrence.

While we focussed our analysis and interpretation of the results around the biopsychosocial model, it is important to consider these findings within other relevant theories and how our results may have focused on different aspects of post treatment wellbeing if these were used. Once example is when considered the Social-Cognitive Transition (SCT) Model of Adjustment which explains the coping mechanisms and subsequent reorientation as a result of physical illness [41]. It describes that physical illness not only results in individual distressful effects but also those of positive effects to aid personal growth. This was similarly demonstrated in the present study by reflection and advocacy for prostate cancer due to their cancer experience. The theory demonstrates the importance of time for reorientation and mental adjustment in the form of emotional processing following an illness event. This forms a key part of individual’s prostate cancer journey to allow the time to adjust and evaluate following diagnosis and treatment of prostate cancer.

Limitations of the existing study do exist, particularly within the diversity of our sample. Prostate cancer disproportionally affects men of African descent; however, our sample unfortunately only contained a small proportion of these individuals [42]. Additionally, studies suggest that men who are identified as single are at higher risk of mortality after radical prostatectomy than married men, and our sample lacked many single men meaning that their experiences may be disproportionally under-represented [43]. This is similar for homosexual men with low representation in our sample, who are unfortunately known to have substantial problems after treatment due to the impacts of erectile dysfunction and varying relationship dynamics, but cannot be fully investigated in the present study [44]. Also, although, unlike previous studies combining all treatment groups, the present study focused on lived experiences of men undergoing radical treatment, there was still heterogeneity within this group. While all patients underwent treatment with curative intent, there are still considerable differences between those undergoing surgery and radiotherapy with regard to treatment and post treatment experiences which should be considered by urologists and oncologists in their clinics when translating these results to their population. Lastly, due to the COVID-19 pandemic interviews were carried out online. Whilst known to be an effective interviewing modality it is possible this may have affected interviewer and participant rapport and selected against those in lower sociodemographic groups without internet or computer access [45].

Our findings have important clinical implications by identifying specific areas of a patient’s prostate cancer journeys which need particular attention. For instance, at the diagnostic stage, the present study focuses on the nature of patients to information gather to aid decision making with some patients exhibiting regret in reflection of those decisions. Clinicians can offer better support by providing more information on the process, risks, and implications of undergoing radical treatment or through the adjunct use of decision aids, such as the Multifactorial decision support systems (mDSS) which have demonstrated benefit [46]. Additionally, uncertainty and fear of recurrence were two important issues. When considering uncertainty, it is important to not only identify the specific sources of this, but additionally try to alleviate these, through psychoeducation programmes [47]. Similarly, fear of cancer recurrence should be managed using cognitive behavioural therapy which has been demonstrated to be low-cost and effective [48]. Finally, participants repeatedly described the distress a prostate cancer diagnosis had on their partner and family, highlighting the importance of considering the wider network in cancer support services. The benefit of this has been shown in breast cancer patients by cancer rehabilitation programs which offer individual psychotherapy and counselling to patients and their family [49].

In light of the findings of the present study, future research should focus on important specific constructs. For instance, recurrence fear varied between patients receiving radical prostatectomy and those receiving radiotherapy, meaning that more focused studies, both quantitative and qualitative, may allow further clarification of the variation between different forms of radical treatment. In addition, studies assessing specific groups of people who were underrepresented in the present study (single men, homosexuality, black ethnicity), would allow more appreciation of the external factors which may impact mental health outcomes in patients undergoing radical treatment. Finally, whilst we highlight key constructs and stages of patient’s cancer journey which are important in wellbeing, further research is needed to highlight the best ways to address these to allow prevention and treatment as current evidence base is critically low.

## Conclusion

Undergoing radical treatments for prostate cancer has a significant impact on the mental, social and physical wellbeing of individuals. We provide an insight into the lived experiences of these men, highlighting the noteworthy distress, anxiety and uncertainty that can be experienced, particularly at diagnosis. Additionally, we demonstrate the importance of social support and changes that are often undertaken in terms of social planning and physical health after diagnosis. These findings allow for key areas where healthcare professionals can provide additional support, to offer reassurance and assistance in decision making and social support to the partner and family which are part of their journey. Future research should aim to identify best ways to support these men, ensuring a more holistic approach to post treatment care to improve overall quality of life in survivorship care.

## Supporting information

S1 Table Interview topic guide. (DOCX)

S2 Table Interrater agreement of generated codes. (DOCX)

S3 Table Full table of generated codes within their biopsychosocial domains. (DOCX)

## References

[1] Sung H, Ferlay J, Siegel RL, Laversanne M, Soerjomataram I, Jemal A, et al. Global Cancer Statistics 2020: GLOBOCAN Estimates of Incidence and Mortality Worldwide for 36 Cancers in 185 Countries. CA: A Cancer Journal for Clinicians. 2021;71(3):209–49. doi: 10.3322/caac.21660 33538338

[2] De Angelis R, Sant M, Coleman MP, Francisci S, Baili P, Pierannunzio D, et al. Cancer survival in Europe 1999–2007 by country and age: results of EUROCARE—5-a population-based study. Lancet Oncol. 2014;15(1):23–34. Epub 20131205. doi: 10.1016/S1470-2045(13)70546-1 . 24314615

[3] Donovan JL, Hamdy FC, Lane JA, Mason M, Metcalfe C, Walsh E, et al. Patient-Reported Outcomes after Monitoring, Surgery, or Radiotherapy for Prostate Cancer. New England Journal of Medicine. 2016;375(15):1425–37. doi: 10.1056/NEJMoa1606221 . 27626365 PMC5134995

[4] De Sousa A, Sonavane S, Mehta J. Psychological aspects of prostate cancer: a clinical review. Prostate Cancer and Prostatic Diseases. 2012;15(2):120–7. doi: 10.1038/pcan.2011.66 22212706

[5] Brunckhorst O, Hashemi S, Martin A, George G, Van Hemelrijck M, Dasgupta P, et al. Depression, anxiety, and suicidality in patients with prostate cancer: a systematic review and meta-analysis of observational studies. Prostate Cancer Prostatic Dis. 2021;24(2):281–9. Epub 20200925. doi: 10.1038/s41391-020-00286-0 . 32978524

[6] Eymech O, Brunckhorst O, Deacon M, James C, Bowie J, Dasgupta P, et al. The Impact of Radical Prostatectomy on the Social Wellbeing of Prostate Cancer Survivors. A Qualitative Meta-Synthesis. 2022. doi: 10.1111/ecc.13630 35754206 PMC11497296

[7] Halpern MT, Argenbright KE. Evaluation of effectiveness of survivorship programmes: how to measure success? Lancet Oncol. 2017;18(1):e51–e9. doi: 10.1016/S1470-2045(16)30563-0 . 28049577

[8] Richards M, Corner J, Maher J. The National Cancer Survivorship Initiative: new and emerging evidence on the ongoing needs of cancer survivors. British Journal of Cancer. 2011;105(1):S1–S4. doi: 10.1038/bjc.2011.416 22048027 PMC3251952

[9] Engel GL. The clinical application of the biopsychosocial model. American Journal of Psychiatry. 1980;137(5):535–44. doi: 10.1176/ajp.137.5.535 . 7369396

[10] Adler RH. Engel’s biopsychosocial model is still relevant today. Journal of psychosomatic research. 2009;67(6):607–11. doi: 10.1016/j.jpsychores.2009.08.008 19913665

[11] Wade DT, Halligan PW. The biopsychosocial model of illness: a model whose time has come. Clin Rehabil. 2017;31(8):995–1004. doi: 10.1177/0269215517709890 . 28730890

[12] van de Wal M, van Oort I, Schouten J, Thewes B, Gielissen M, Prins J. Fear of cancer recurrence in prostate cancer survivors. Acta Oncologica. 2016;55(7):821–7. doi: 10.3109/0284186X.2016.1150607 26935517

[13] James C, Brunckhorst O, Eymech O, Stewart R, Dasgupta P, Ahmed K. Fear of cancer recurrence and PSA anxiety in patients with prostate cancer: a systematic review. Support Care Cancer. 2022;30(7):5577–89. Epub 20220201. doi: 10.1007/s00520-022-06876-z . 35106656 PMC9135793

[14] Maheu C, Hébert M, Louli J, Yao TR, Lambert S, Cooke A, et al. Revision of the fear of cancer recurrence cognitive and emotional model by Lee-Jones et al with women with breast cancer. Cancer Rep (Hoboken). 2019;2(4):e1172. Epub 20190404. doi: 10.1002/cnr2.1172 . 32721129 PMC7941532

[15] Bowie J, Brunckhorst O, Stewart R, Dasgupta P, Ahmed K. Body image, self-esteem, and sense of masculinity in patients with prostate cancer: a qualitative meta-synthesis. Journal of Cancer Survivorship. 2022;16(1):95–110. doi: 10.1007/s11764-021-01007-9 33963973 PMC8881246

[16] Levy A, Cartwright T. Men’s strategies for preserving emotional well-being in advanced prostate cancer: An interpretative phenomenological analysis. Psychology & Health. 2015;30(10):1164–82. doi: 10.1080/08870446.2015.1040016 25871263

[17] Keyes CLM. Social well-being. Social psychology quarterly. 1998:121–40.

[18] Cicognani E, Klimstra T, Goossens L. Sense of community, identity statuses, and loneliness in adolescence: A cross-national study on Italian and Belgian youth. Journal of Community Psychology. 2014;42(4):414–32.

[19] Kamen C, Mustian KM, Heckler C, Janelsins MC, Peppone LJ, Mohile S, et al. The association between partner support and psychological distress among prostate cancer survivors in a nationwide study. J Cancer Surviv. 2015;9(3):492–9. Epub 20150121. doi: 10.1007/s11764-015-0425-3 . 25603949 PMC4510042

[20] Luckenbaugh AN, Wallis CJD, Huang L-C, Wittmann D, Klaassen Z, Zhao Z, et al. Association between Treatment for Localized Prostate Cancer and Mental Health Outcomes. Journal of Urology. 2022;207(5):1029–37. doi: 10.1097/JU.0000000000002370 34978488 PMC9933911

[21] Krahn MD, Bremner KE, Tomlinson G, Naglie G. Utility and health-related quality of life in prostate cancer patients 12 months after radical prostatectomy or radiation therapy. Prostate Cancer Prostatic Dis. 2009;12(4):361–8. Epub 20090901. doi: 10.1038/pcan.2009.32 . 19901935

[22] Jarzemski P, Brzoszczyk B, Popiołek A, Stachowicz-Karpińska A, Gołota S, Bieliński M, et al. Cognitive function, depression, and anxiety in patients undergoing radical prostatectomy with and without adjuvant treatment. Neuropsychiatr Dis Treat. 2019;15:819–29. Epub 20190405. doi: 10.2147/NDT.S200501 . 31040681 PMC6454999

[23] O’Brien BC, Harris IB, Beckman TJ, Reed DA, Cook DA. Standards for reporting qualitative research: a synthesis of recommendations. Acad Med. 2014;89(9):1245–51. doi: 10.1097/ACM.0000000000000388 . 24979285

[24] Neubauer BE, Witkop CT, Varpio L. How phenomenology can help us learn from the experiences of others. Perspectives on Medical Education. 2019;8(2):90–7. doi: 10.1007/s40037-019-0509-2 30953335 PMC6468135

[25] Low J. A Pragmatic Definition of the Concept of Theoretical Saturation. Sociological Focus. 2019;52(2):131–9. doi: 10.1080/00380237.2018.1544514

[26] Malterud K, Siersma VD, Guassora AD. Sample Size in Qualitative Interview Studies: Guided by Information Power. Qual Health Res. 2016;26(13):1753–60. Epub 20160710. doi: 10.1177/1049732315617444 . 26613970

[27] Britten N. Qualitative Research: Qualitative interviews in medical research. BMJ. 1995;311(6999):251–3. doi: 10.1136/bmj.311.6999.251 7627048 PMC2550292

[28] Kallio H, Pietilä AM, Johnson M, Kangasniemi M. Systematic methodological review: developing a framework for a qualitative semi-structured interview guide. J Adv Nurs. 2016;72(12):2954–65. Epub 20160623. doi: 10.1111/jan.13031 . 27221824

[29] Braun V, Clarke V. Using thematic analysis in psychology. Qualitative Research in Psychology. 2006;3(2):77–101.

[30] O’Connor C, Joffe H. Intercoder Reliability in Qualitative Research: Debates and Practical Guidelines. International Journal of Qualitative Methods. 2020;19:1609406919899220. doi: 10.1177/1609406919899220

[31] Pessoa ASG, Harper E, Santos IS, Gracino MCdS. Using Reflexive Interviewing to Foster Deep Understanding of Research Participants’ Perspectives. International Journal of Qualitative Methods. 2019;18:1609406918825026. doi: 10.1177/1609406918825026

[32] Gannon K, Guerro-Blanco M, Patel A, Abel P. Re-constructing masculinity following radical prostatectomy for prostate cancer. The Aging Male. 2010;13(4):258–64. doi: 10.3109/13685538.2010.487554 21067475

[33] Watts S, Leydon G, Birch B, Prescott P, Lai L, Eardley S, et al. Depression and anxiety in prostate cancer: a systematic review and meta-analysis of prevalence rates. BMJ Open. 2014;4(3):e003901. doi: 10.1136/bmjopen-2013-003901 24625637 PMC3963074

[34] Miller LE. Sources of uncertainty in cancer survivorship. J Cancer Surviv. 2012;6(4):431–40. Epub 20120720. doi: 10.1007/s11764-012-0229-7 . 22815086

[35] Guan T, Santacroce SJ, Chen DG, Song L. Illness uncertainty, coping, and quality of life among patients with prostate cancer. Psychooncology. 2020;29(6):1019–25. Epub 20200313. doi: 10.1002/pon.5372 . 32128938 PMC7440775

[36] Maly RC, Umezawa Y, Leake B, Silliman RA. Mental health outcomes in older women with breast cancer: impact of perceived family support and adjustment. Psychooncology. 2005;14(7):535–45. doi: 10.1002/pon.869 . 15493064

[37] Ruiz-Rodríguez I, Hombrados-Mendieta I, Melguizo-Garín A, Martos-Méndez MJ. The Importance of Social Support, Optimism and Resilience on the Quality of Life of Cancer Patients. Front Psychol. 2022;13:833176. Epub 20220309. doi: 10.3389/fpsyg.2022.833176 . 35356348 PMC8959607

[38] Kronenwetter C, Weidner G, Pettengill E, Marlin R, Crutchfield L, McCormac P, et al. A Qualitative Analysis of Interviews of Men with Early Stage Prostate Cancer: The Prostate Cancer Lifestyle Trial. Journal of Urology. 2006;175(2):675-. doi: 10.1016/S0022-5347(05)00275-2 15815179

[39] Newby TA, Graff JN, Ganzini LK, McDonagh MS. Interventions that may reduce depressive symptoms among prostate cancer patients: a systematic review and meta-analysis. Psycho-Oncology. 2015;24(12):1686–93. doi: 10.1002/pon.3781 25753507

[40] Anderson E, McCormack M. Inclusive Masculinity Theory: overview, reflection and refinement. Journal of Gender Studies. 2018;27(5):547–61. doi: 10.1080/09589236.2016.1245605

[41] Brennan J. Adjustment to cancer—coping or personal transition? Psychooncology. 2001;10(1):1–18. doi: 10.1002/1099-1611(200101/02)10:1&lt;1::aid-pon484&gt;3.0.co;2-t . 11180573

[42] Rebbeck TR. Prostate Cancer Genetics: Variation by Race, Ethnicity, and Geography. Semin Radiat Oncol. 2017;27(1):3–10. Epub 20160826. doi: 10.1016/j.semradonc.2016.08.002 . 27986209 PMC5175208

[43] Khan S, Nepple KG, Kibel AS, Sandhu G, Kallogjeri D, Strope S, et al. The association of marital status and mortality among men with early-stage prostate cancer treated with radical prostatectomy: insight into post-prostatectomy survival strategies. Cancer Causes Control. 2019;30(8):871–6. Epub 20190618. doi: 10.1007/s10552-019-01194-y . 31214808 PMC6739072

[44] Sandfort TGM, de Graaf R, Bijl RV, Schnabel P. Same-Sex Sexual Behavior and Psychiatric Disorders: Findings From the Netherlands Mental Health Survey and Incidence Study (NEMESIS). Archives of General Psychiatry. 2001;58(1):85–91. doi: 10.1001/archpsyc.58.1.85 11146762

[45] Foley G. Video-based online interviews for palliative care research: A new normal in COVID-19? Palliat Med. 2021;35(3):625–6. Epub 20210121. doi: 10.1177/0269216321989571 . 33475026 PMC8072795

[46] van Wijk Y, Halilaj I, van Limbergen E, Walsh S, Lutgens L, Lambin P, et al. Decision Support Systems in Prostate Cancer Treatment: An Overview. Biomed Res Int. 2019;2019:4961768. Epub 20190606. doi: 10.1155/2019/4961768 . 31281840 PMC6590598

[47] Guan T, Qan’ir Y, Song L. Systematic review of illness uncertainty management interventions for cancer patients and their family caregivers. Support Care Cancer. 2021;29(8):4623–40. Epub 20210125. doi: 10.1007/s00520-020-05931-x . 33495851 PMC8236440

[48] Burm R, Thewes B, Rodwell L, Kievit W, Speckens A, van de Wal M, et al. Long-term efficacy and cost-effectiveness of blended cognitive behavior therapy for high fear of recurrence in breast, prostate and colorectal Cancer survivors: follow-up of the SWORD randomized controlled trial. BMC Cancer. 2019;19(1):462. Epub 20190516. doi: 10.1186/s12885-019-5615-3 . 31096934 PMC6524293

[49] Weis J. Psychosocial Care for Cancer Patients. Breast Care (Basel). 2015;10(2):84–6. doi: 10.1159/000381969 . 26195935 PMC4463789

