# Supplementary material for: Undergoing radical treatment for prostate cancer and its impact on wellbeing: A qualitative study exploring men’s experiences
Source: PLoS One. 2022 Dec 16;17(12):e0279250. doi: 10.1371/journal.pone.0279250 (PMC9757548; doi:10.1371/journal.pone.0279250)
Supplement: S1 Table — (DOCX) [file pone.0279250.s001.docx]

| **Topic** | **Core Questions/Statements** | **Follow-Up Questions/Prompts if Unanswered** |
| --- | --- | --- |
| **Introduction** | Who we are and aims of the study.  Ensure PIS read and consent form completed  ‘Will start recording now if ok’ | ‘If at any point you do not want to continue or wish to take a break please let us know’  ‘Do you have any questions for us before we begin?’ |
| **Demographics, Cancer and Social History** | Age  Time Since Diagnosis  Treatments Received  Civil Status  Occupation  Previous/current mental health diagnosis |  |
| **Patient Perception of Wellbeing after Diagnosis** | ‘What impact did a prostate cancer diagnosis have on your life?’ | What do you understand by the term ‘wellbeing’?  ‘What do you think this term should encompass?’  ‘How was your ‘wellbeing’ affected?’  ‘Which aspects do you feel are most important for your quality of life?’  ‘Can you give me any examples of particular aspects you found difficult?’ |
| **Mental Wellbeing I**  **(Mood)** | ‘In the context of your cancer diagnosis what does the term ‘mental wellbeing’ mean to you?’  ‘How did your diagnosis and treatment impact your mental wellbeing?’ | ‘Do you see any difference between ‘mental wellbeing’ and ‘mental health’?  ‘How was your mood after your diagnosis’  ‘Was anxiety ever something which impacted you at any point?’  ‘Was there anything specific that made you anxious or low in mood?’  Can you give me an example of when things were at their worst?’ |
| **Mental Wellbeing II**  **(Peripheral concepts if not covered already)** | ‘Outside of mood, some people find that other aspects of their mental wellbeing can be impacted. Was there ever anything else which affected you?’ | ‘How did you feel in your body after your diagnosis or treatment?’  ‘Was there any change in how you saw yourself?’  ‘What was your experience with worrying about your cancer itself?  ‘Can you give any examples? ‘ |
| **Physical Wellbeing** | ‘How was your physical health after your diagnosis or treatment?’  ‘Sex life can be changed after being diagnosed, what was your experience?’ | ‘What bothered you the most?’  ‘Have any of these impacted other parts of your life?’  ‘Do you think these impacted your mental wellbeing or alternatively the other way around?’  ‘Can you give any examples?’ |
| **Social Wellbeing** | ‘How did prostate cancer impact your social or family life?’ | ‘Did anything change with your family and friends during this time?’  ‘How was your working life at this time?’ (If employed)  ‘How about everyday life and jobs at home, did this change?’ |
| **Completion** | ‘Thank you very much for taking part in the study, your answers have been very useful’ | ‘Is there anything else you think I missed or should know?’  ‘Do you have any questions for me?’ |
