# Supplementary material for: Undergoing radical treatment for prostate cancer and its impact on wellbeing: A qualitative study exploring men’s experiences
Source: PLoS One. 2022 Dec 16;17(12):e0279250. doi: 10.1371/journal.pone.0279250 (PMC9757548; doi:10.1371/journal.pone.0279250)
Supplement: S2 Table — (DOCX) [file pone.0279250.s002.docx]

| **Code** | **Kappa** | **Agreement** |
| --- | --- | --- |
| A Trade off | 0.00 | 99.82 |
| Acceptance | 1.00 | 100.00 |
| Ageing Body | 1.00 | 100.00 |
| Anger | 0.80 | 99.97 |
| Anxiety | 0.45 | 99.60 |
| Becoming an Advocate | 1.00 | 100.00 |
| Biopsy Issues | 0.90 | 99.95 |
| Bleak Future | 1.00 | 100.00 |
| Body Image | 0.31 | 99.89 |
| Bowel Symptoms | 1.00 | 100.00 |
| Burden | 1.00 | 100.00 |
| Careful Choice of Language | 0.28 | 99.77 |
| Catheter Experience | 0.79 | 99.96 |
| Changing Perspective | 0.56 | 99.64 |
| Concealment from Family | 0.79 | 99.93 |
| Death, Dying and Own Mortality | 1.00 | 100.00 |
| Decision Regret | 0.68 | 99.85 |
| Depression or Low Mood | 0.63 | 99.87 |
| Disappointment | 0.55 | 99.91 |
| Faith in God | 0.36 | 99.86 |
| Family Distress | 0.39 | 99.86 |
| Family Support | 1.00 | 100.00 |
| Fatigue | 0.93 | 99.99 |
| Fear of Cancer Recurrence or Progression | 0.88 | 99.83 |
| Fear of Invasive Procedures | 0.63 | 99.86 |
| Fear of Repeating Family History | 0.75 | 99.92 |
| Feeling Lucky | 0.67 | 99.77 |
| Fitness and Exercise | 1.00 | 100.00 |
| Friend’s Support | 0.95 | 99.95 |
| Get on With It | 0.99 | 99.98 |
| Get Rid of It | 1.00 | 100.00 |
| Grateful | 0.57 | 99.79 |
| Healthcare Trust | 1.00 | 100.00 |
| Healthy Eating | 1.00 | 100.00 |
| Hobbies Restricted | 0.52 | 99.97 |
| Hormone Symptoms | 0.37 | 99.60 |
| Incontinence | 0.74 | 99.90 |
| Increased Awareness of Body | 0.38 | 99.81 |
| Information Gathering | 0.99 | 99.98 |
| Isolation | 0.00 | 99.81 |
| Lack of Control or Maintaining Control | 1.00 | 100.00 |
| Loneliness | 1.00 | 100.00 |
| Loss of Body Ownership | 0.00 | 99.95 |
| Loss of Libido | 0.87 | 99.93 |
| Mourning Lost Sex Life | 1.00 | 100.00 |
| Open and Honest | 1.00 | 100.00 |
| Optimistic | 0.81 | 99.79 |
| Pandemic Uncertainty | 0.78 | 99.87 |
| Partner Anxiety | 0.73 | 99.77 |
| Partner Pressure | 1.00 | 100.00 |
| Partner Support | 1.00 | 100.00 |
| Physical Restriction | 0.59 | 99.95 |
| Planning | 1.00 | 100.00 |
| Positive Thoughts | 0.48 | 99.82 |
| Pragmatic | 1.00 | 100..00 |
| Previous Knowledge | 0.88 | 99.92 |
| PSA Anxiety | 1.00 | 100.00 |
| Reflection | 0.87 | 99.88 |
| Relief | 1.00 | 100.00 |
| Renegotiating Relationship and Intimacy | 0.00 | 99.90 |
| Scared | 0.12 | 99.77 |
| Searching Alternative Medical Treatments | 1.00 | 100.00 |
| Seeking Reassurance | 0.93 | 99.93 |
| Setting Goals | 1.00 | 100.00 |
| Sexual Dysfunction | 0.86 | 99.77 |
| Shared Decision Making | 1.00 | 100.00 |
| Shock | 0.81 | 99.76 |
| Storage Symptoms | 0.54 | 99.82 |
| Straining Relationship | 0.00 | 99.81 |
| Strengthening Relationship | 0.00 | 99.86 |
| Support Groups and Services | 0.98 | 99.98 |
| Survivors Support | 1.00 | 100.00 |
| Threat to Masculinity | 1.00 | 100.00 |
| Treatment Discomfort | 0.76 | 99.85 |
| Treatment Uncertainty | 0.90 | 99.99 |
| Uncertainty | 0.13 | 99.80 |
| Unknown Future | 0.86 | 99.92 |
| Voiding Symptoms | 1.00 | 100.00 |
| Why Me | 0.79 | 99.96 |
| Work Pressures | 0.65 | 99.92 |
| Worry over Family Future | 0.00 | 99.87 |
| Worry over Treatment Outcomes | 1.00 | 100.00 |
