# Supplementary material for: Undergoing radical treatment for prostate cancer and its impact on wellbeing: A qualitative study exploring men’s experiences
Source: PLoS One. 2022 Dec 16;17(12):e0279250. doi: 10.1371/journal.pone.0279250 (PMC9757548; doi:10.1371/journal.pone.0279250)
Supplement: S3 Table — (DOCX) [file pone.0279250.s003.docx]

| **Codes** | | |
| --- | --- | --- |
| **Physical Wellbeing** | | |
| Ageing Body | Biopsy Issues | Bowel Symptoms |
| Catheter Experience | Fatigue | Fitness and Exercise |
| Healthy Eating | Hormone Symptoms | Incontinence |
| Loss of Libido | Physical Restriction | Sexual Dysfunction |
| Storage Symptoms | Treatment Discomfort | Voiding Symptoms |
| **Psychological Wellbeing** | | |
| A Trade off | Acceptance | Anger |
| Anxiety | Careful Choice of Language | Bleak Future |
| Body Image | Changing Perspective | Death, Dying and Own Mortality |
| Decision Regret | Depression or Low Mood | Disappointment |
| Faith in God | Fear of Cancer Recurrence or Progression | Fear of Invasive Procedures |
| Fear of Repeating Family History | Feeling Lucky | Get on with it |
| Get Rid of it | Grateful | Increased Awareness of Body |
| Information Gathering | Isolation | Lack of Control or Maintaining Control |
| Loneliness | Loss of Body Ownership | Mourning Lost Sex Life |
| Optimistic | Positive Thoughts | Pragmatic |
| Previous Knowledge | PSA Anxiety | Reflection |
| Relief | Scared | Searching Alternative Medical Treatments |
| Seeking Reassurance | Setting Goals | Shock |
| Threat to Masculinity | Treatment Uncertainty | Uncertainty |
| Unknown Future | Why Me | Worry over Treatment Outcomes |
| **Social Wellbeing** | | |
| Becoming an Advocate | Burden | Concealment from Family |
| Family Distress | Family Support | Friend’s Support |
| Healthcare Trust | Hobbies Restricted | Open and Honest |
| Pandemic Uncertainty | Partner Anxiety | Partner Pressure |
| Partner Support | Planning | Renegotiating Relationship and Intimacy |
| Shared Decision Making | Straining Relationship | Strengthening Relationship |
| Support Groups and Services | Survivors Support | Work Pressures |
| Worry over Family Future |  |  |
