# Supplementary material for: Undergoing radical treatment for prostate cancer and its impact on wellbeing: A qualitative study exploring men’s experiences
Source: PLoS One. 2022 Dec 16;17(12):e0279250. doi: 10.1371/journal.pone.0279250 (PMC9757548; doi:10.1371/journal.pone.0279250)
Supplement: Attachment — Submitted filename: Structured Responses to Reviewer Comments.docx [file pone.0279250.s004.docx]

**Structured Responses to Reviewer Comments**

**Reviewer #1:**

Vyas and colleagues performed a qualitative study analyzing the impact of radical treatment on mental wellbeing of men with prostate cancer. They found key themes of an uncertain future and social planning which is related to that of radical treatment. This demonstrated the profound impact on quality of life within all biological, psychological, and social aspects.

Thank you for the opportunity to review this article entitled “Undergoing radical treatment for prostate cancer and wellbeing: a qualitative study exploring men’s experiences”. The manuscript is well-written and a pleasure to read. However, I have some comments for consideration:
Many thanks for your review of the manuscript and your positive comments

Title
The title is somewhat unclear. It can be read as: Undergoing radical treatment for ....wellbeing? The title should be changed to be more precise.

Thank you for highlighting this confusion. We agree with this and have therefore changed the title of the manuscript to be more precise - Undergoing radical treatment for prostate cancer and its impact on wellbeing: a qualitative study exploring men’s experiences (Line 1-2).

Result
Many themes are presented in the result-section. It seems a bit overwhelming list and quite descriptive. Is it possible to merge some of them?

We have reviewed the themes throughout and have merged two of the themes to attempt to reduce the number of themes. This includes ‘Diagnostic Blow’ and ‘the Search for Clarity’ as these themes have close association and resemblance in the cancer journey of participants. We have amended the figures for this accordingly. However, on review of the remaining themes we believed that combining these would either remove the order of the patient journey experienced for mental wellbeing themes, or instead were not closely linked enough to be able to link together. Similarly, by removing themes we believe we would be reducing the quality of the overall wellbeing impact described within the manuscript. We believed that this would have reduced the overall quality of the manuscript and have therefore kept the remaining themes separate.

Theory
A good qualitative article presents relevant theories to shed light on the findings and how these are interpreted and discussed. In this article the results that are presented and discussed just by the biopsychosocial model, rather than not seen in the light of any other theory. For example, they could incorporate theory of disruption, coping, reorientation…... According to Bradbury-Jones, C., Taylor, J., & Herber, O. (2014). How theory is used and articulated in qualitative research: development of a new typology. Soc Sci Med, 120, 135-141., lack of theory in qualitative research undermines its quality.

Many thanks for this point. We agree that inclusion of other theory is important and can shed light to different focus of the results if these had been considered. We have therefore included a section within the discussion section where we consider our findings within the Social-Cognition Transition (SCT) Model of Adjustment which highlights this key characteristic of coping mechanisms and reorientation due to illnesses of a physical nature. It demonstrates the importance of time for processing the emotions because of prostate cancer diagnosis and treatment (Lines 512-523). We hope this gives some greater depth to this section of the manuscript.

The term significant is repeated numerous times in the article. It is a central term in quantitative research applying statistical methods. In qualitative research, synonyms are often better suited.

Many thanks for highlighting this, we have altered this to various synonyms throughout the manuscript including: substantial, profound, important and meaningful to avoid repetition.

**Reviewer #2**:

Thank you for inviting me to review this important topic which is investigating lived experiences of men following treatment for prostate cancer through qualitative research.
Minor suggestions as follows:

Thank you for your time reviewing the manuscript and providing the insightful comments.

Methods:
Omit in the inclusion criteria: Participants were eligible regardless of ethnicity, civil status, and sexual orientation.

We agree and have removed this line from the methods section.

Row 159 - Change to BSc not Bachelor

This has been altered.

Results:
191 - most were married (except two) - suggest change this to percentage to flow with rest of this paragraph. Suggest introducing Table 1 in row 194.

Percentages has been included in this section and the introduction of Table 1 has been included where mentioned.

Can the authors further define occupation (working - is it part/full time etc).

Unfortunately, this data was not collected during the initial data collection interviews and is no longer available to be collected from participants at this time. We have therefore not been able to include this level of detail within the occupation of participants.

Can length of time from TX completion be reported for (1) for those who had surgery; (2) and those who had radiotherapy?

We have added this information within Table 1 with a column for ‘Time since treatment completion (months)’ for both surgery and radiotherapy participants.

Limitations to include the mix of patients who had surgery and radiotherapy which creates heterogenity. Is the sample recruited representative of the patients in your clinic (worth commenting on)

Thank you for this point. We agree and have included a discussion surrounding this within the discussion section (Lines 532-539) to highlight the heterogeneity within ‘radical treatment’ group. Although all participants underwent treatment with curative intent, we have further acknowledged there are differences in treatment and post-treatment experiences of men undergoing either surgery or radiotherapy. This is identified as a key aspect for urologists and oncologists when interpreting these findings and translating this to their population of patients.

Minor Typos throughout need to be checked

We have reviewed the manuscript throughout and have amended spelling and grammar mistakes identified.
