# Supplementary material for: Undergoing radical treatment for prostate cancer and its impact on wellbeing: A qualitative study exploring men’s experiences
Source: PLoS One. 2022 Dec 16;17(12):e0279250. doi: 10.1371/journal.pone.0279250 (PMC9757548; doi:10.1371/journal.pone.0279250)
Supplement: Peer review history

# Peer review: Decision Letter 0

**DOI:** 10.1371/journal.pone.0279250.r001

**Authors:** Adetayo Olorunlana

4 Nov 2022

PONE-D-22-24731Undergoing radical treatment for prostate cancer and wellbeing: a qualitative study exploring men’s experiencesPLOS ONE

Dear Dr. Brunckhorst,

Thank you for submitting your manuscript to PLOS ONE. After careful consideration, we feel that it has merit but does not fully meet PLOS ONE’s publication criteria as it currently stands. Therefore, we invite you to submit a revised version of the manuscript that addresses the points raised during the review process.

Please submit your revised manuscript by Dec 19 2022 11:59PM. If you will need more time than this to complete your revisions, please reply to this message or contact the journal office at plosone@plos.org. When you're ready to submit your revision, log on to https://www.editorialmanager.com/pone/ and select the 'Submissions Needing Revision' folder to locate your manuscript file.

Please include the following items when submitting your revised manuscript:

- A rebuttal letter that responds to each point raised by the academic editor and reviewer(s). You should upload this letter as a separate file labeled 'Response to Reviewers'.
- A marked-up copy of your manuscript that highlights changes made to the original version. You should upload this as a separate file labeled 'Revised Manuscript with Track Changes'.
- An unmarked version of your revised paper without tracked changes. You should upload this as a separate file labeled 'Manuscript'.

If you would like to make changes to your financial disclosure, please include your updated statement in your cover letter. Guidelines for resubmitting your figure files are available below the reviewer comments at the end of this letter.

If applicable, we recommend that you deposit your laboratory protocols in protocols.io to enhance the reproducibility of your results. Protocols.io assigns your protocol its own identifier (DOI) so that it can be cited independently in the future. For instructions see: https://journals.plos.org/plosone/s/submission-guidelines#loc-laboratory-protocols. Additionally, PLOS ONE offers an option for publishing peer-reviewed Lab Protocol articles, which describe protocols hosted on protocols.io. Read more information on sharing protocols at https://plos.org/protocols?utm_medium=editorial-email&utm_source=authorletters&utm_campaign=protocols.

We look forward to receiving your revised manuscript.

Kind regards,

Adetayo Olorunlana, Ph.D.

Academic Editor

PLOS ONE

Journal Requirements:

When submitting your revision, we need you to address these additional requirements.

1. Please ensure that your manuscript meets PLOS ONE's style requirements, including those for file naming. The PLOS ONE style templates can be found at 

https://journals.plos.org/plosone/s/file?id=wjVg/PLOSOne_formatting_sample_main_body.pdf and 

https://journals.plos.org/plosone/s/file?id=ba62/PLOSOne_formatting_sample_title_authors_affiliations.pdf

2. Please include captions for your Supporting Information files at the end of your manuscript, and update any in-text citations to match accordingly. Please see our Supporting Information guidelines for more information: http://journals.plos.org/plosone/s/supporting-information. 

3. Please review your reference list to ensure that it is complete and correct. If you have cited papers that have been retracted, please include the rationale for doing so in the manuscript text, or remove these references and replace them with relevant current references. Any changes to the reference list should be mentioned in the rebuttal letter that accompanies your revised manuscript. If you need to cite a retracted article, indicate the article’s retracted status in the References list and also include a citation and full reference for the retraction notice.

[Note: HTML markup is below. Please do not edit.]

Reviewers' comments:

Reviewer's Responses to Questions

**Comments to the Author**

1. Is the manuscript technically sound, and do the data support the conclusions?

The manuscript must describe a technically sound piece of scientific research with data that supports the conclusions. Experiments must have been conducted rigorously, with appropriate controls, replication, and sample sizes. The conclusions must be drawn appropriately based on the data presented. 

Reviewer #1: Yes

Reviewer #2: Yes

**********

2. Has the statistical analysis been performed appropriately and rigorously? 

Reviewer #1: N/A

Reviewer #2: Yes

**********

3. Have the authors made all data underlying the findings in their manuscript fully available?

The PLOS Data policy requires authors to make all data underlying the findings described in their manuscript fully available without restriction, with rare exception (please refer to the Data Availability Statement in the manuscript PDF file). The data should be provided as part of the manuscript or its supporting information, or deposited to a public repository. For example, in addition to summary statistics, the data points behind means, medians and variance measures should be available. If there are restrictions on publicly sharing data—e.g. participant privacy or use of data from a third party—those must be specified.

Reviewer #1: Yes

Reviewer #2: Yes

**********

4. Is the manuscript presented in an intelligible fashion and written in standard English?

PLOS ONE does not copyedit accepted manuscripts, so the language in submitted articles must be clear, correct, and unambiguous. Any typographical or grammatical errors should be corrected at revision, so please note any specific errors here.

Reviewer #1: Yes

Reviewer #2: Yes

**********

5. Review Comments to the Author

Please use the space provided to explain your answers to the questions above. You may also include additional comments for the author, including concerns about dual publication, research ethics, or publication ethics. (Please upload your review as an attachment if it exceeds 20,000 characters)

Reviewer #1: Thank you for the opportunity to review this article entitled “Undergoing radical treatment for prostate cancer and wellbeing: a qualitative study exploring men’s experiences”. The manuscript is well-written and a pleasure to read. However, I have some comments for consideration.

Title

The title is somewhat unclear. It can be read as: Undergoing radical treatment for ....wellbeing? The title should be changed to be more precise.

Result

Many themes are presented in the result-section. It seems a bit overwhelming list and quite descriptive. Is it possible to merge some of them?

Theory

A good qualitative article presents relevant theories to shed light on the findings and how these are interpreted and discussed. In this article the results that are presented and discussed just by the biopsychosocial model, rather than not seen in the light of any other theory. For example, they could incorporate theory of disruption, coping, reorientation…... According to Bradbury-Jones, C., Taylor, J., & Herber, O. (2014). How theory is used and articulated in qualitative research: development of a new typology. Soc Sci Med, 120, 135-141., lack of theory in qualitative research undermines its quality.

The term significant is repeated numerous times in the article. It is a central term in quantitative research applying statistical methods. In qualitative research, synonyms are often better suited.

Reviewer #2: thank you for inviting me to review this important topic which is investigating lived experiences of men following treatment for prostate cancer through qualitative research.

Minor suggestions as follows:

Methods:

Omit in the inclusion criteria: Participants were eligible regardless of ethnicity, civil status, and sexual orientation.

Row 159 - Change to BSc not Bachelor

Results:

191 - most were married (except two) - suggest change this to percentage to flow with rest of this paragraph. Suggest introducing Table 1 in row 194.

Can the authors further define occupation (working - is it part/full time etc).

Can length of time from TX completion be reported for (1) for those who had surgery; (2) and those who had radiotherapy?

Limitations to include the mix of patients who had surgery and radiotherapy which creates heterogenity. Is the sample recruited representative of the patients in your clinic (worth commenting on)

Minor Typos throughout need to be checked

**********

6. PLOS authors have the option to publish the peer review history of their article (what does this mean?). If published, this will include your full peer review and any attached files.

If you choose “no”, your identity will remain anonymous but your review may still be made public.

**Do you want your identity to be public for this peer review?** For information about this choice, including consent withdrawal, please see our Privacy Policy.

Reviewer #1: No

Reviewer #2: No

**********

[NOTE: If reviewer comments were submitted as an attachment file, they will be attached to this email and accessible via the submission site. Please log into your account, locate the manuscript record, and check for the action link "View Attachments". If this link does not appear, there are no attachment files.]

While revising your submission, please upload your figure files to the Preflight Analysis and Conversion Engine (PACE) digital diagnostic tool, https://pacev2.apexcovantage.com/. PACE helps ensure that figures meet PLOS requirements. To use PACE, you must first register as a user. Registration is free. Then, login and navigate to the UPLOAD tab, where you will find detailed instructions on how to use the tool. If you encounter any issues or have any questions when using PACE, please email PLOS at figures@plos.org. Please note that Supporting Information files do not need this step.

# Author comment: Author response to Decision Letter 0

**DOI:** 10.1371/journal.pone.0279250.r002

28 Nov 2022

Structured Responses to Reviewer Comments 

Reviewer #1: 

Vyas and colleagues performed a qualitative study analyzing the impact of radical treatment on mental wellbeing of men with prostate cancer. They found key themes of an uncertain future and social planning which is related to that of radical treatment. This demonstrated the profound impact on quality of life within all biological, psychological, and social aspects.

Thank you for the opportunity to review this article entitled “Undergoing radical treatment for prostate cancer and wellbeing: a qualitative study exploring men’s experiences”. The manuscript is well-written and a pleasure to read. However, I have some comments for consideration:

Many thanks for your review of the manuscript and your positive comments 

Title

The title is somewhat unclear. It can be read as: Undergoing radical treatment for ....wellbeing? The title should be changed to be more precise.

Thank you for highlighting this confusion. We agree with this and have therefore changed the title of the manuscript to be more precise - Undergoing radical treatment for prostate cancer and its impact on wellbeing: a qualitative study exploring men’s experiences (Line 1-2).

Result

Many themes are presented in the result-section. It seems a bit overwhelming list and quite descriptive. Is it possible to merge some of them?

We have reviewed the themes throughout and have merged two of the themes to attempt to reduce the number of themes. This includes ‘Diagnostic Blow’ and ‘the Search for Clarity’ as these themes have close association and resemblance in the cancer journey of participants. We have amended the figures for this accordingly. However, on review of the remaining themes we believed that combining these would either remove the order of the patient journey experienced for mental wellbeing themes, or instead were not closely linked enough to be able to link together. Similarly, by removing themes we believe we would be reducing the quality of the overall wellbeing impact described within the manuscript. We believed that this would have reduced the overall quality of the manuscript and have therefore kept the remaining themes separate. 

Theory

A good qualitative article presents relevant theories to shed light on the findings and how these are interpreted and discussed. In this article the results that are presented and discussed just by the biopsychosocial model, rather than not seen in the light of any other theory. For example, they could incorporate theory of disruption, coping, reorientation…... According to Bradbury-Jones, C., Taylor, J., & Herber, O. (2014). How theory is used and articulated in qualitative research: development of a new typology. Soc Sci Med, 120, 135-141., lack of theory in qualitative research undermines its quality.

Many thanks for this point. We agree that inclusion of other theory is important and can shed light to different focus of the results if these had been considered. We have therefore included a section within the discussion section where we consider our findings within the Social-Cognition Transition (SCT) Model of Adjustment which highlights this key characteristic of coping mechanisms and reorientation due to illnesses of a physical nature. It demonstrates the importance of time for processing the emotions because of prostate cancer diagnosis and treatment (Lines 512-523). We hope this gives some greater depth to this section of the manuscript. 

The term significant is repeated numerous times in the article. It is a central term in quantitative research applying statistical methods. In qualitative research, synonyms are often better suited.

Many thanks for highlighting this, we have altered this to various synonyms throughout the manuscript including: substantial, profound, important and meaningful to avoid repetition.

Reviewer #2: 

Thank you for inviting me to review this important topic which is investigating lived experiences of men following treatment for prostate cancer through qualitative research.

Minor suggestions as follows:

Thank you for your time reviewing the manuscript and providing the insightful comments.

Methods:

Omit in the inclusion criteria: Participants were eligible regardless of ethnicity, civil status, and sexual orientation.

We agree and have removed this line from the methods section. 

Row 159 - Change to BSc not Bachelor

This has been altered.

Results:

191 - most were married (except two) - suggest change this to percentage to flow with rest of this paragraph. Suggest introducing Table 1 in row 194.

Percentages has been included in this section and the introduction of Table 1 has been included where mentioned.

Can the authors further define occupation (working - is it part/full time etc).

Unfortunately, this data was not collected during the initial data collection interviews and is no longer available to be collected from participants at this time. We have therefore not been able to include this level of detail within the occupation of participants. 

Can length of time from TX completion be reported for (1) for those who had surgery; (2) and those who had radiotherapy?

We have added this information within Table 1 with a column for ‘Time since treatment completion (months)’ for both surgery and radiotherapy participants.

Limitations to include the mix of patients who had surgery and radiotherapy which creates heterogenity. Is the sample recruited representative of the patients in your clinic (worth commenting on)

Thank you for this point. We agree and have included a discussion surrounding this within the discussion section (Lines 532-539) to highlight the heterogeneity within ‘radical treatment’ group. Although all participants underwent treatment with curative intent, we have further acknowledged there are differences in treatment and post-treatment experiences of men undergoing either surgery or radiotherapy. This is identified as a key aspect for urologists and oncologists when interpreting these findings and translating this to their population of patients.

Minor Typos throughout need to be checked

We have reviewed the manuscript throughout and have amended spelling and grammar mistakes identified.

Attachment Submitted filename: Structured Responses to Reviewer Comments.docx Click here for additional data file.

# Editor report: Decision Letter 1

**DOI:** 10.1371/journal.pone.0279250.r003

**Authors:** Adetayo Olorunlana

4 Dec 2022

Undergoing radical treatment for prostate cancer and its impact on wellbeing: a qualitative study exploring men’s experiences

PONE-D-22-24731R1

Dear Dr. Brunckhorst,

We’re pleased to inform you that your manuscript has been judged scientifically suitable for publication and will be formally accepted for publication once it meets all outstanding technical requirements.

Within one week, you’ll receive an e-mail detailing the required amendments. When these have been addressed, you’ll receive a formal acceptance letter and your manuscript will be scheduled for publication.

An invoice for payment will follow shortly after the formal acceptance. To ensure an efficient process, please log into Editorial Manager at http://www.editorialmanager.com/pone/, click the 'Update My Information' link at the top of the page, and double check that your user information is up-to-date. If you have any billing related questions, please contact our Author Billing department directly at authorbilling@plos.org.

If your institution or institutions have a press office, please notify them about your upcoming paper to help maximize its impact. If they’ll be preparing press materials, please inform our press team as soon as possible -- no later than 48 hours after receiving the formal acceptance. Your manuscript will remain under strict press embargo until 2 pm Eastern Time on the date of publication. For more information, please contact onepress@plos.org.

Kind regards,

Adetayo Olorunlana, Ph.D.

Academic Editor

PLOS ONE

Additional Editor Comments (optional):

Reviewers' comments:

# Editor report: Acceptance letter

**DOI:** 10.1371/journal.pone.0279250.r004

**Authors:** Adetayo Olorunlana

8 Dec 2022

PONE-D-22-24731R1 

Undergoing radical treatment for prostate cancer and its impact on wellbeing: a qualitative study exploring men’s experiences 

Dear Dr. Brunckhorst:

I'm pleased to inform you that your manuscript has been deemed suitable for publication in PLOS ONE. Congratulations! Your manuscript is now with our production department. 

If your institution or institutions have a press office, please let them know about your upcoming paper now to help maximize its impact. If they'll be preparing press materials, please inform our press team within the next 48 hours. Your manuscript will remain under strict press embargo until 2 pm Eastern Time on the date of publication. For more information please contact onepress@plos.org.

If we can help with anything else, please email us at plosone@plos.org. 

Thank you for submitting your work to PLOS ONE and supporting open access. 

Kind regards, 

PLOS ONE Editorial Office Staff

on behalf of

Associate Professor Adetayo Olorunlana 

Academic Editor

PLOS ONE
